# Venlafaxine Attenuated the Cognitive and Memory Deficit in Mice Exposed to Isoflurane Alone

**DOI:** 10.3389/fneur.2021.591223

**Published:** 2021-02-23

**Authors:** Liang Li, Chunhai Zhang

**Affiliations:** ^1^Department of Orthopedics, Shenzhen Hospital, South Medical University, Shenzhen, China; ^2^Department of Thyroid Surgery, China-Japan Union Hospital of Jilin University, Changchun, China

**Keywords:** POCD, GABAA receptors α5, neuroinflammation, Y maze, memory

## Abstract

Post-operative cognitive dysfunction (POCD) is a common complication during the post-operative period. It affects the recovery time of the patient after surgery and the stay time in hospital, which causes a great deal of burden to patients and families emotionally and financially. However, there is no specific and effective treatment available for this disorder. Recent study indicated exposure to general anesthetics contributed to POCD by triggering gamma-amino butyric acid type A (GABA_A_) receptors hyperactivities that persisted even the anesthetic compounds have been eliminated. Here, we investigated the antidepressant, venlafaxine (VLX), in a mouse model of POCD and studied whether VLX attenuated the cognitive dysfunction of mice exposed to general anesthetic, isoflurane (ISO). We found that ISO significantly induced an increased surface expression of the GABA_A_ receptor subunit, α5, in the hippocampus of the mice. However, VLX treatment reduced the increase in α5 subunit expression. Meanwhile, we found the expression levels of interleukin (IL)-1β, tumor necrosis factor alpha (TNF-α), and IL-6 in the brains of mice exposed to ISO were significantly increased. However, VLX could prevent the increase in these cytokines. We also investigated the memory deficit of these mice by using a Y maze behavioral test. Mice with ISO exposure showed decreased alternation performance that could be prevented by the VLX treatment. Collectively, our results here are in line with the previous findings that α5 subunit plays an important role of the formation of POCD, but VLX may be a promising candidate compound for the treatment of POCD.

## Introduction

Post-operative cognitive dysfunction (POCD) is featured with cognitive decline, such as memory and executive functions after surgeries, including thyroid cancers, especially in elderly patients. It could last days, weeks, and months even longer than several years (1). Statistical data suggest that the proportion of POCD after surgery in elderly patients is as high as 10% at 3 months after surgery (2). The persistent cognitive dysfunction adversely affects the life quality of patients, such as long time bedridden stage and recovery time, which could cause heavy burden to the patients and their families (3). Multiple factors are considered to predispose POCD, such as aging and other pre-existing medical conditions (4). However, the underlying mechanisms of this disorder remain ambiguous so far. Few options of the treatment are specifically for POCD so far due to lack of the extensive knowledge of this disorder. Recent studies indicated that the type of anesthetics, anesthetic method, and the duration of anesthetics were critical factors that contributed to the development of POCD (3, 5), which suggested that understanding the anesthetics action mechanism might lead to important clinical significance for the prevention and treatment of POCD in the future. Meanwhile, a promising treatment strategy could be developed based on the further understanding of the pathogenesis of POCD. Therefore, the study aimed to explore the etiology of POCD after anesthesia treatment and the possible treatment option of the disorder.

In the central nervous system (CNS), gamma-amino butyric acid type A receptors (GABA_A_Rs) are critical inhibitory receptors, and most general anesthetics are positive allosteric modulators of GABA_A_Rs by increasing the inhibition in the brain, which causes amnesia and loss of consciousness (6). Among them, GABA_A_Rs containing an a5 subunit are relevant in the POCD after exposure to general anesthesia (3, 7). One of the general anesthetics, etomidate, could cause sustained increase in α5GABA_A_R activity and impair memory performance and synaptic plasticity. Furthermore, inhibition of the α5GABA_A_R could prevent the mentioned memory deficits (3). Established studies have suggested that neuroinflammation resulting from anesthesia or surgery significantly contributed to the development of POCD (8). In human, general anesthesia was associated with cognitive dysfunction and the increased expression of interleukin (IL)-6 and tumor necrosis factor alpha (TNF-α), which could be attenuated by dexmedetomidine (9). In rat model, ISO exposure alone could cause the higher expression levels of cytokines in the hippocampus (10). In another pre-clinical study, ISO could boost the release of IL-1β in the hippocampal CA1 region of rats (11). However, the specific relationship between neuroinflammation and POCD treatment is still not profiled yet.

Venlafaxine (VLX) is one of the widely used antidepressants due to its relatively safe profile and efficacy on depressive and anxiety disorders (12). Study also implicated that VLX could improve the cognitive performance in patients and animals (13, 14). These results promoted us to postulate whether VLX could be a potential compound for the treatment of POCD. This assumption was also supported by the fact that VLX has been post-operatively used for the patients in a variety of clinical settings (15, 16). Moreover, VLX has been found to possess neuroprotective effects (17), which could be another important property to support the potential treatment for POCD since POCD was associated with synaptic protein dysfunction (18).

In the present study, we investigated the possible effect of VLX on the prevention of POCD in mice exposed to ISO. We found that ISO could cause decreased spontaneous alternation of mice in a Y maze test that is a behavioral assay of working memory (19), but VLX could effectively protect the mice from the above memory loss. We also explored the underlying mechanism by which VLX could exert these beneficial effects.

## Materials and Methods

### Animals and Drug Treatments

**Figure 1:**
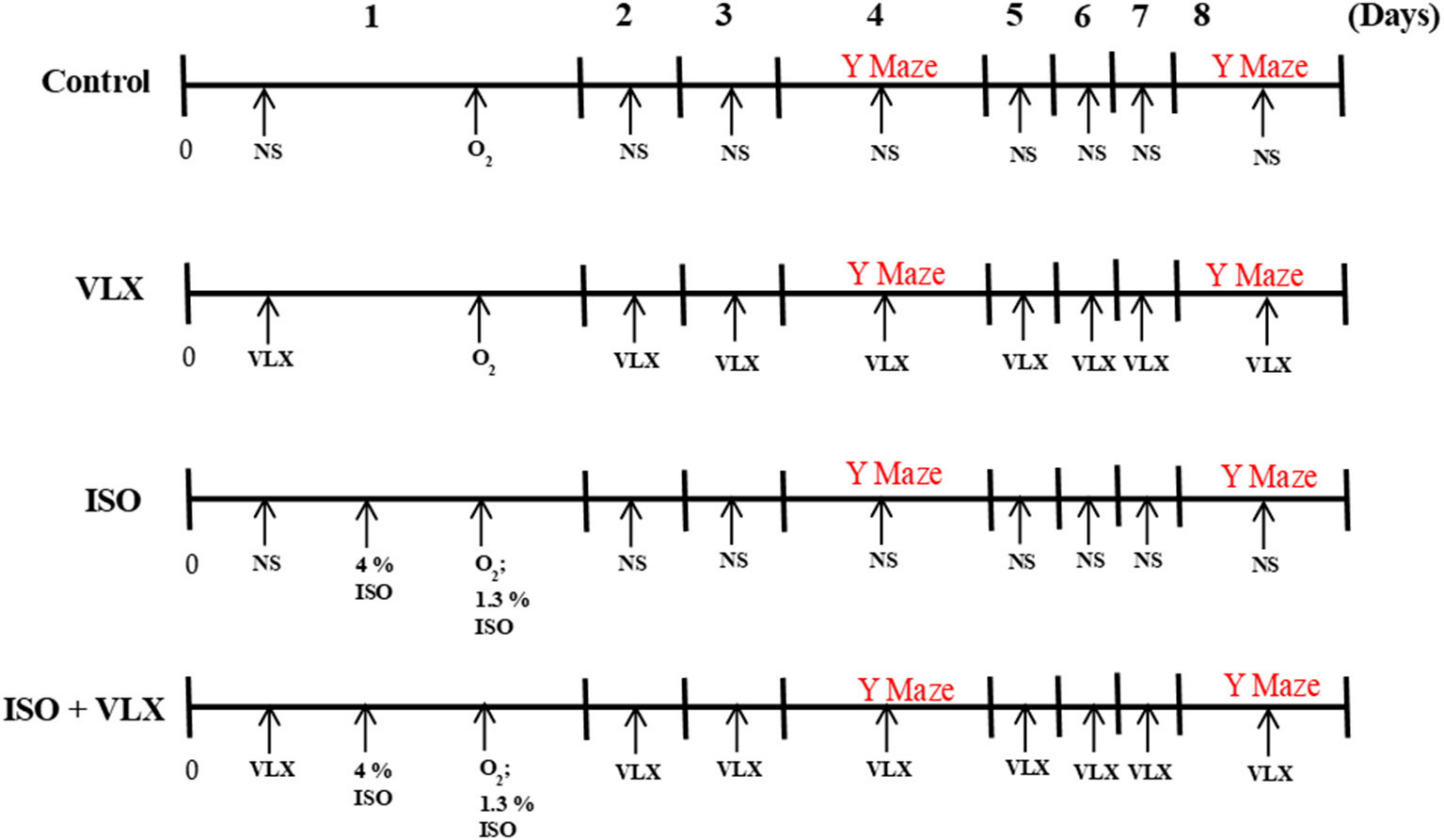
Brief diagram of the experimental protocol. NS, normal saline; O_2_, 100% oxygen; VLX, venlafaxine; ISO, isoflurane.

All procedures involving animals were approved by the Experimental Animal Ethics Committee of China–Japan Union Hospital of Jilin University, Jilin Province, China. Male C57BL/6 mice at the age of 12 weeks old were used in the present experiments. Mice were kept in the standard laminal-flow cabinets with pathogen-free conditions. Mice were randomly grouped into multiple groups: control group, venlafaxine alone group (VLX), ISO alone group (ISO), and ISO plus venlafaxine group (ISO + VLX). VLX was purchased from Sigma-Aldrich and dissolved in normal saline. For the ISO treatment, the mice were anesthetized with inhalation of 4% ISO for the induction phase and then 1.3% for 3 h in a chamber on a heating pad to keep body temperature with 100% oxygen (20). The breath and heartbeat of the mice were closely monitored by an experimenter. VLX (16 mg/kg) and normal saline were administrated intraperitoneally on a daily basis till the day when behavioral test was completed. For the control group and VLX alone group, mice were either injected with normal saline or VLX intraperitoneally and then went through 3 h in chambers with 100% oxygen but without ISO on the first day. The concentration of VLX used here was adapted from the previous report (21). A brief diagram of the experimental design was presented (Figure 1).

### Tissue Collection

Mice were sacrificed on the day when the behavioral tests were completed. After perfusion with 0.9% normal saline, the hippocampal tissues from the mice brains were collected for the determination of protein concentration with Western blot, ELISA, and biotinylation.

### Y Maze

Working memory was assessed in a Y maze apparatus consisting of three arms, which was a 1-day test. The Y maze test was carried out for 3 days and 1 week later after the anesthesia. Each arm of the Y maze was 35 cm long, 25 cm high, and 10 cm wide and positioned at an equal angle as previously reported (22). The three arms were labeled as A, B, and C. During the test, mice were put at the end of one arm randomly and allowed to explore freely the arms in 8 min. We manually recorded the sequence of arm entries during the test. The alternation and total arms entries were analyzed and compared among groups.

### Western Blot

To measure the protein expression level of p-STAT3 and STAT3, tissues from the hippocampus of mice were lysed with radioimmunoprecipitation assay (RIPA) buffer and then centrifuged at 12,000 g at cold condition. The supernatant containing equal amount of total protein was separated in sodium dodecyl sulfate–polyacrylamide gel electrophoresis (SDS-PAGE) gel for 2 h. After transferring to polyvinylidene fluoride (PVDF) membranes and blocking with 5% bovine serum albumin (BSA) in Tris-buffered saline with Tween 20 (TBST) buffer, the membranes were incubated with primary antibodies of p-STAT3 (1:1,000, Cell Signaling, #9145), STAT3 (1:1,000, Cell Signaling, #9132), and β-actin (1:4,000, Santa Cruz Biotechnology, sc-517582) in cold room for overnight and then in secondary antibodies for 2 more hours at room temperature after washing with TBST. Chemiluminescence with enhanced chemiluminescence (ECL) solution was used to visualize the signal from the membranes. The expression level of each protein in statistical analysis was presented as the relative ratio compared to the internal control protein, β-actin.

### ELISA

Cytokines were measured with the commercial ELISA kits (Thermo Scientific) according to manufacturer's protocols, respectively. Briefly, the total protein was isolated from hippocampal tissues as above mentioned in the Western blot, and the supernatant was used for ELISA assay after concentration was determined. All samples were assayed with duplication. Standard curve for each cytokine was established with their standard samples provided in the kits. The values of these cytokines were normalized as a relative ratio compared to the total protein.

### Cell-Surface Biotinylation

Cell-surface biotinylation was carried out as previously reported (3). Briefly, 350-μm coronal slices were prepared with a vibratome and then immediately transferred to ice-cold artificial cerebrospinal fluid (ACSF) oxygenation with 95% O_2_/5% CO_2_. The slices were then incubated twice in ice-cold 0.75 mg/ml NHS-SS-biotin (Thermo Scientific) for 45 min. Then, slices were washed several times to stop the excess biotin reaction followed by adding lysis buffer with protease inhibitors. Hi-Capacity NeutrAvidin beads (Thermo Scientific) was used to incubate supernatant for 16–18 h at 4°C to pull down and elute the proteins with elution buffer. The protein concentration was determined, and equal amount protein was subjected to SDS-PAGE analysis as Western blot assay. GABA_A_ receptor α5 subunit antibody (1:1,000, Millipore, ab9678), β-actin antibody (1:4,000, Santa Cruz Biotechnology, sc-517582), and anti-Na+/K+ ATPase antibody (1:500, Abcam, ab58475) were used for Western blot analysis. The expression of α5 was normalized to its respective loading controls in the surface (NKA) and total (β-actin). The purity of extracted surface protein was determined by probing the presence of β-actin with antibody.

### Statistical Analysis

All statistical analysis was completed with Prism software. Data were expressed as the mean ± SEM. The significance of differences was determined by two-way ANOVA, followed by Tukey's *post-hoc* test for multiple comparisons. A *p* < 0.05 was deemed to show statistical significance.

## Results

### VLX Ameliorated the Memory Deficit of Mice Exposed to ISO in a Y Maze Test

**Figure 2:**
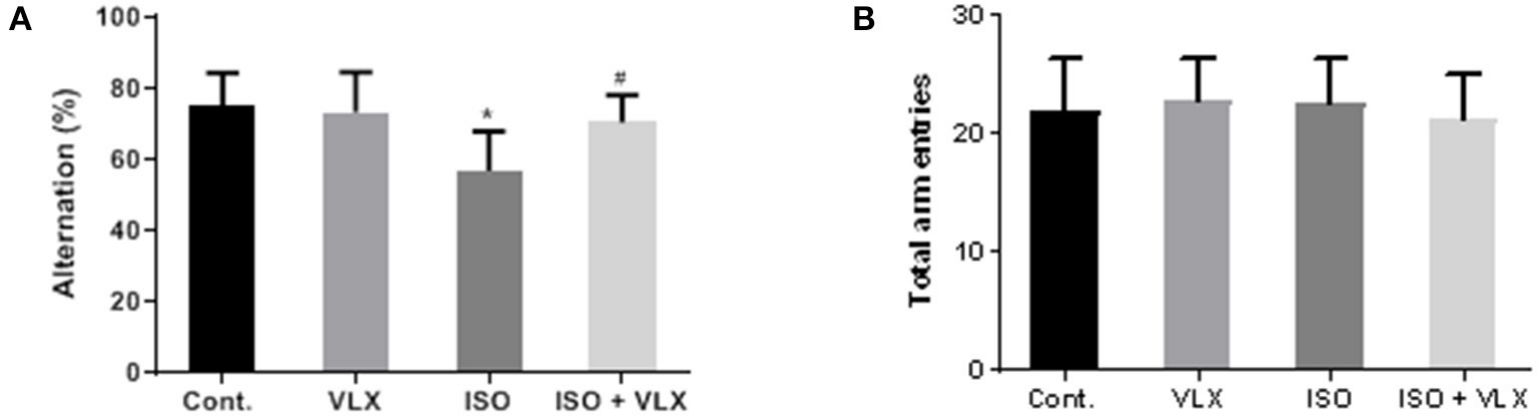
Venlafaxine (VLX) ameliorated the memory deficit in Y maze test of mice exposed to isoflurane (ISO) (day 3). **(A)** The effect of VLX on the alternation of mice in Y maze test. **(B)** The effect of VLX on the total arm entries of mice in Y maze test. Data were expressed as mean ± SEM. ^*^p < 0.05 compared to control; ^#^*p* < 0.05 compared to ISO, *n* = 12.

**Figure 3:**
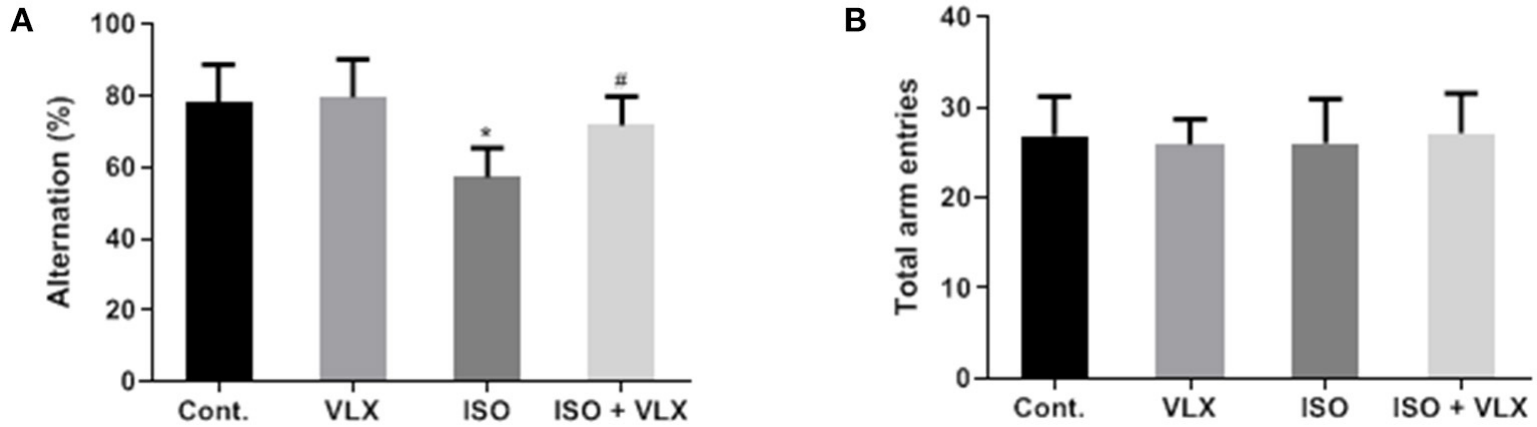
Venlafaxine (VLX) attenuated the memory deficit in Y maze test of mice exposed to isoflurane (ISO) (1 week). **(A)** The effect of VLX on the alternation of mice in Y maze test. **(B)** The effect of VLX on the total arm entries of mice in Y maze test. Data were expressed as mean ± SEM. ^*^p < 0.05 compared to control; ^#^*p* < 0.05 compared to ISO, *n* = 12.

To test the protective effects of VLX on memory deficit, we carried out a Y maze behavioral test to assess the status of working memory of the mice with ISO treatment. We carried out the test on day 3 and 1 week after the anesthesia. On day 3, we found that ISO caused a lower alternation performance in the transition of A, B, and C arms, which indicated compromised working memory of these mice. However, the deterioration of cognitive function could be significantly prevented when we employed the VLX treatment to these mice. Two-way ANOVA analysis demonstrated that ISO [*F*_1, 44_ = 14.12, *p* < 0.001] and VLX [*F*_1, 44_ = 4.297, *p* < 0.05] exerted a significant change in the alternation performance. There was an interaction between ISO and VLX [*F*_1, 44_ = 7.738, *p* < 0.01] in day 3. A *post-hoc* analysis indicated that the alternation behavior in the ISO-treated mice was less than that of the control mice (*p* < 0.001), and VLX significantly prevented the reduction in the alternation performance (*p* < 0.01) (Figure 2A). Interestingly, the treatments of VLX and ISO did not affect the total arm entries among all groups, which suggested that the changes in alternation were not due to the alteration of the motor function of these mice (Figure 2B). Moreover, the total arm entries in all groups were comparable, which indicated neither ISO nor VLX treatment was able to affect the motor function of mice (Figure 2B). On the time point of 1 week after anesthesia, we observed the similar results. Two-way ANOVA analysis demonstrated that ISO [*F*_1, 44_ = 28.18, p < 0.0001] and VLX [*F*_1, 44_ = 8.531, *p* < 0.01] exerted a significant change in the alternation performance. There was an interaction between ISO and VLX [*F*_1, 44_ = 5.787, *p* < 0.05] in 1 week. A *post-hoc* analysis indicated that the alternation behavior in the ISO-treated mice was less than that of the control mice (*p* < 0.0001), and VLX significantly prevented the reduction in the alternation performance (*p* < 0.01) (Figure 3A). ISO and VLX showed no effect on the total arm entries (Figure 3B). These above results suggested that ISO exposure could adversely affect the working memory in mice, but VLX could rescue the mice from the ISO-induced memory deficit.

### VLX Reduced the Phosphorylation of STAT3 in Mice Exposed to ISO

**Figure 4:**
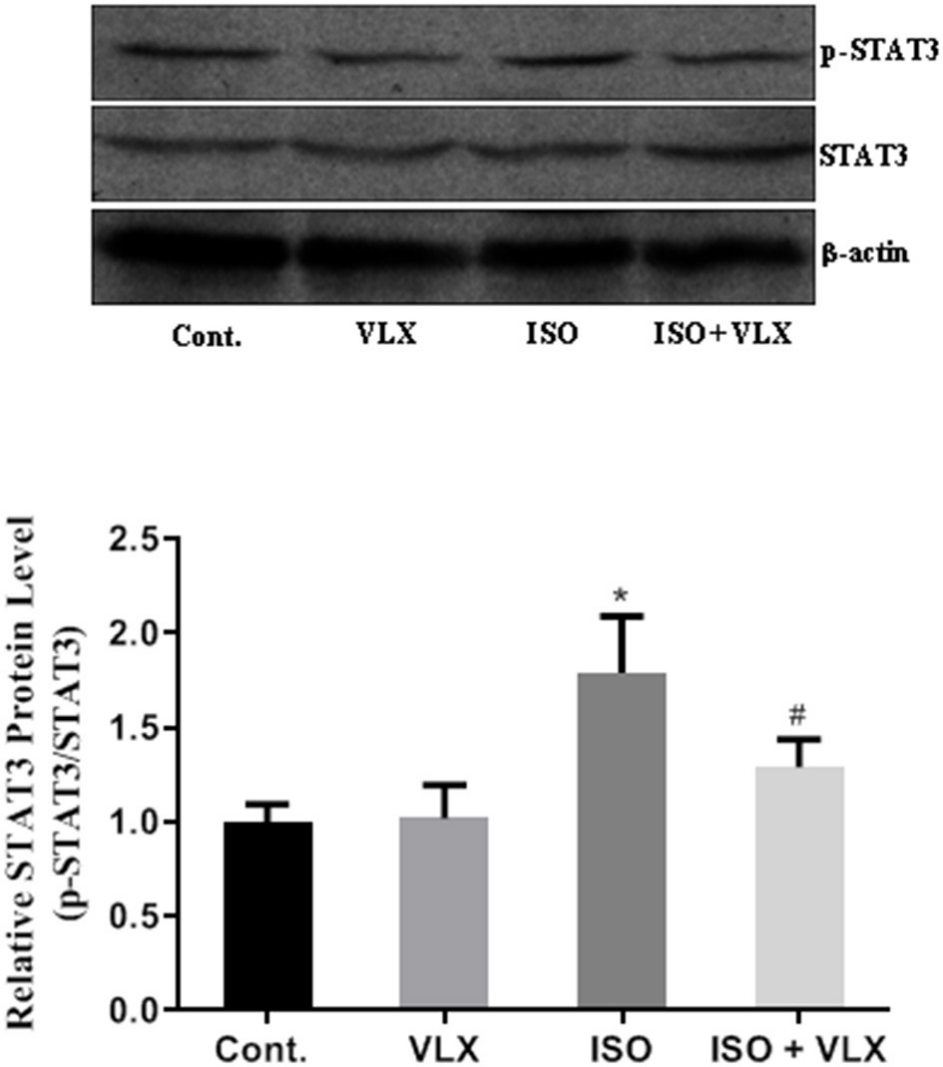
Venlafaxine (VLX) reduced the phosphorylation of STAT3 in mice exposed to isoflurane (ISO) in Western blot assay. Data were expressed as mean ± SEM. ^*^p < 0.05 compared to control; ^#^*p* < 0.05 compared to ISO, *n* = 6.

JAK2/STAT3 axis has been considered as an important signaling pathway that was disturbed in the memory loss under certain circumstance (23). In addition, STAT3 was one of the key players in neuroinflammatory response that was found to be responsible for memory and cognitive impairment in certain extent (24, 25). Therefore, we performed Western blot assay to measure whether there was a change in STAT3 expression level in the mouse hippocampus. Our results demonstrated that ISO exposure did not affect the expression level of total STAT3 protein but significantly increased the expression level of phosphorylated STAT3 (p-STAT3). Importantly, we found that VLX could inhibit the upregulation of p-STAT3 expression level. Two-way ANOVA analysis demonstrated that ISO [*F*_1, 20_ = 44.60, *p* < 0.0001] and VLX [*F*_1, 20_ = 9.073, p < 0.01] exerted a significant change in the protein level of p-STAT3. There was an interaction between ISO and VLX [*F*_1, 20_ = 10.79, *p* < 0.01] on the change of p-STAT3 protein. A *post-hoc* analysis indicated that p-STAT3 protein in the ISO-treated mice was higher than that of the control mice (*p* < 0.0001), and VLX significantly prevented the increase in the p-STAT3 protein (*p* < 0.01) (Figure 4). These above results indicated that ISO might activate the STAT3 signaling pathway via enhancing its phosphorylation, and VLX could exert its beneficial effect by preventing the phosphorylation.

### VLX Attenuated the Levels of Cytokines in the Hippocampus of Mice Exposed to ISO

**Figure 5:**
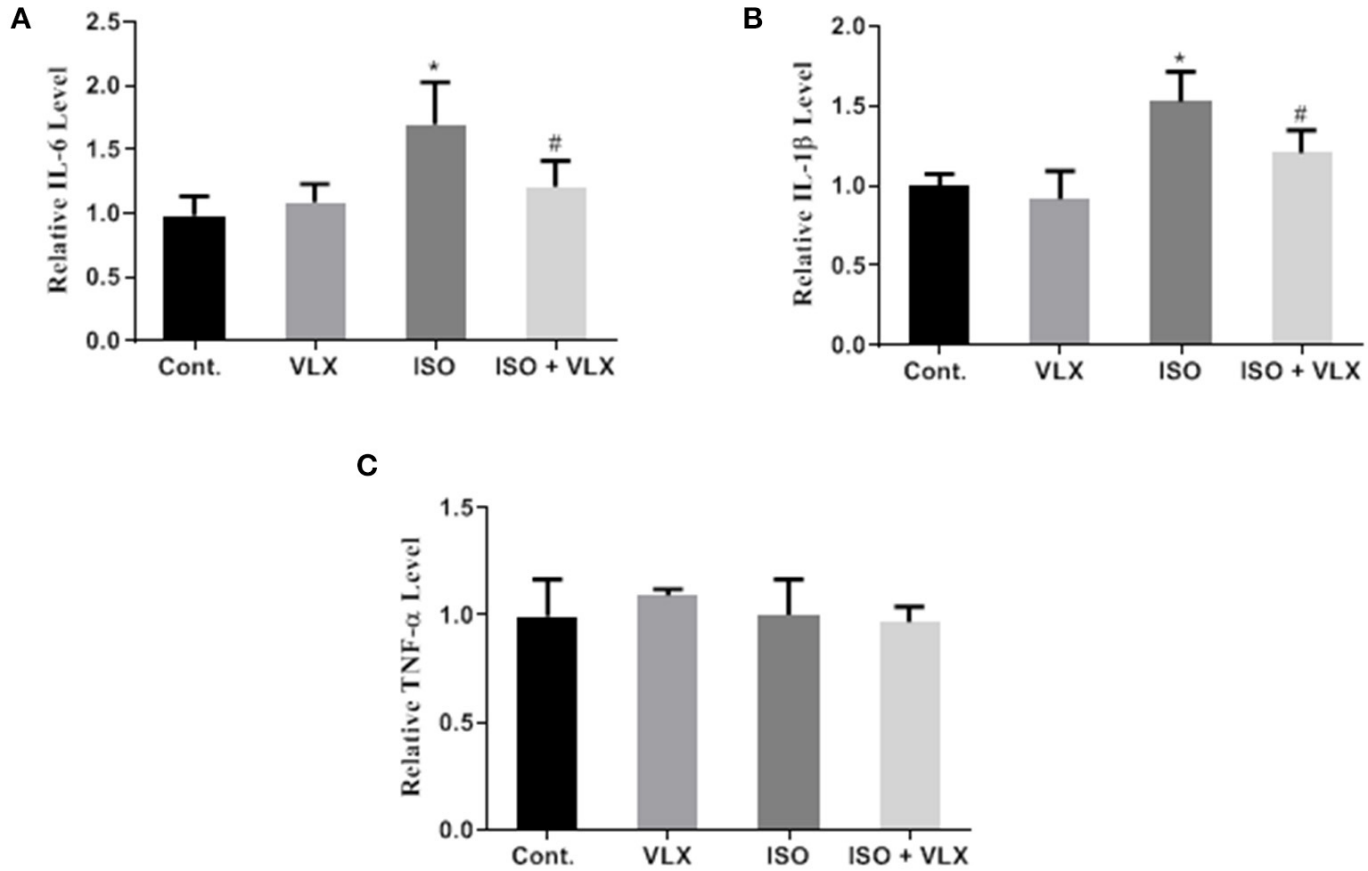
Venlafaxine (VLX) attenuated the products of cytokine in hippocampus of mice exposed to isoflurane (ISO) in ELISA assay. **(A)** The effect of VLX on the expression of IL-6 of mice exposed to ISO. **(B)** The effect of VLX on the expression of IL-1β of mice exposed to ISO. **(C)** The effect of VLX on the expression of tumor necrosis factor alpha (TNF-α) of mice exposed to ISO. Data were expressed as mean ± SEM. ^*^p < 0.05 compared to control; ^#^*p* < 0.05 compared to ISO, *n* = 7.

Based on the above findings and the fact that STAT3 activation was closely associated with neuroinflammation (26), we postulated that VLX might regulate the inflammatory response in the brains of these mice. We carried out ELISA assays to measure the cytokine products in the hippocampal tissues of the mice. We found that the levels of IL-1β and IL-6 were significantly increased by ISO, but there was no change in TNF-α in these mice (Figure 5), which suggested that IL-1β and IL-6 not TNF-α were activated by ISO treatment. For IL-6, two-way ANOVA analysis demonstrated that ISO [*F*_1, 24_ = 24.80, *p* < 0.0001] and VLX [*F*_1, 24_ = 5.140, *p* < 0.05] exerted a significant change in the IL-6 protein level. There was an interaction between ISO and VLX [*F*_1, 24_ = 12.32, *p* < 0.01] on the changes of IL-6 protein level. A *post-hoc* analysis indicated that the IL-6 protein level in the ISO-treated mice was higher than that of control mice (*p* < 0.0001), and VLX significantly prevented the increase in the IL-6 protein level (*p* < 0.01) (Figure 5A). For IL-1β, two-way ANOVA analysis demonstrated that ISO [*F*_1, 24_ = 50.62, *p* < 0.0001] and VLX [*F*_1, 24_ = 12.48, *p* < 0.01] exerted a significant change in the IL-1β protein level. There was an interaction between ISO and VLX [*F*_1, 24_ = 4.271, *p* < 0.05] on the changes in IL-1β protein level. A *post-hoc* analysis indicated that the IL-1β protein level in the ISO-treated mice was higher than that of the control mice (*p* < 0.0001), and VLX significantly prevented the increase in the IL-1β protein level (*p* < 0.01) (Figure 5B). For TNF-α, ISO and VLX showed no effect on its protein expression level (Figure 5C). These results implied that VLX might exert anti-inflammatory effects in these mice exposed to ISO by inhibiting the levels of IL-1β and IL-6 in their hippocampus.

### VLX Decreased the Surface Expression of GABA_A_ Receptor α5 in Mice Exposed to ISO

**Figure 6:**
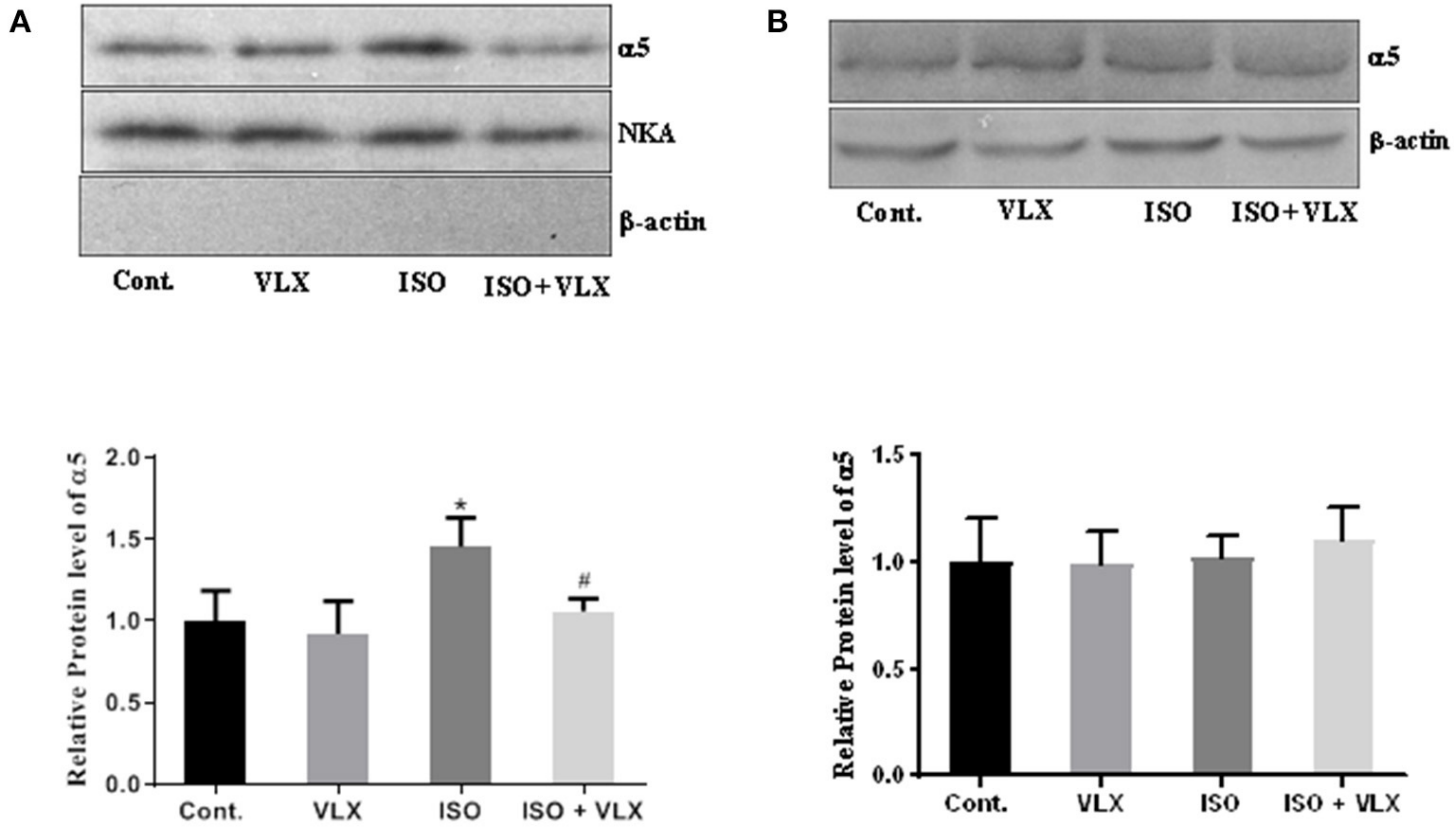
Venlafaxine (VLX) decreased the surface expression of GABA_A_ receptor α5 in mice exposed to isoflurane (ISO). **(A)** The effect of VLX on the surface expression of α5 receptor in mice exposed to ISO. **(B)** The effect of VLX on the total expression of α5 receptor in mice exposed to ISO. Data were expressed as mean ± SEM. ^*^p < 0.05 compared to control; ^#^*p* < 0.05 compared to ISO, *n* = 6.

Increased surface expression of GABA_A_ receptor α5 was shown to be responsible for the changes in tonic current that was considered as the molecular mechanism of memory impairment in POCD (3). Here, we investigated whether VLX regulated the response process of α5 receptor in the mice stimulated with ISO. In line with the previous findings, we found that ISO could increase the surface expression level of α5 receptor (Figure 6A), which suggested that ISO might influence tonic current and then affect the memory and cognitive deficits as suggested by a previous study (3). As anticipated, VLX could decrease the expression level of α5 receptor on cell surface of the hippocampus in mice with ISO. Two-way ANOVA analysis demonstrated that ISO [*F*_1, 16_ = 16.11, *p* < 0.01] and VLX [*F*_1, 16_ = 10.40, *p* < 0.01] exerted a significant change in the surface expression level of α5. There was an interaction between ISO and VLX [*F*_1, 16_ = 4.573, *p* < 0.05] on the changes in the surface expression level of α5. A *post-hoc* analysis indicated that the surface expression level of α5 in the ISO-treated mice was higher than that of the control mice (*p* < 0.01), and VLX significantly prevented the increase in the surface expression level of α5 (*p* < 0.01) (Figure 6A). There was no β-actin expression detected in the surface protein samples as shown in Figure 6A. Meanwhile, we also employed the Western blot analysis in the total protein to probe the expression level of α5. We found that ISO and VLX showed no effect on total protein of α5 (Figure 6B), which suggested that ISO did not affect the total protein expression of α5 but increased the surface expression only.

## Discussion

POCD happens frequently following major surgery. The incidence of this disorder is around one-third in major surgeries and much higher in elderly patients. However, there is no specific treatment option for it yet.

In the present study, we found that the antidepressant, VLX, could be a promising candidate compound to handle the disorder. Cotreatment of VLX could prevent the cognitive and memory deterioration in the mice exposed to anesthetic compound, isoflurane. Our study validated the previous findings by highlighting the involvement of GABA_A_ receptor α5 in the anesthetics-induced POCD. The mouse model used here has been verified by several recent studies from other groups by using single dose of etomidate, which caused memory and cognitive impairment, POCD (3, 7). More importantly, we also found that the protective effects of VLX could last at least 1 week (Figure 3). Mice are often affected by the acute sedative effect of ISO, and to further investigate whether the effect could last longer, we carried out the Y maze test on day 3 and 1 week after the anesthesia. The average total arm entries among all groups were less in day 3 than 1 week (Figures 2, 3). We postulated that it might be due to the acute influence of ISO on the mouse motivation. The repeated effects shown on both day 3 and 1 week from our results made us to claim the protective effects of VLX in the POCD mouse model. Although there existed controversial conclusion of the effects of VLX on memory and cognitive function in clinical studies (27–29), findings from some early stage preclinical studies favored the beneficial roles of VLX (14, 30). Especially, a recent study revealed that VLX rescued the memory loss in the Y maze test in a demyelination mouse model (31), which promoted us to evaluate the possible role of VLX in POCD with the Y maze test.

The dose of VLX used in the present study was based on the previous reports. One report claimed that higher doses (75, 100, and 150 mg/kg) of venlafaxine could lower the threshold of convulsion in animals and suggested the use of 25 and 50 mg/kg for animal study (32). Another study found that venlafaxine exerted stimulant effect on mouse activity in a locomotor test from the dose of 16 mg/kg (33). More importantly, a study indicated that 16 mg/kg could significantly affect the function of the hippocampus (21). The common mechanism of VLX is blocking the reuptake of serotonin and norepinephrine in CNS and leaving more these neurotransmitters in the synapse. In the dose of 16 mg/kg, VLX could reduce the noradrenaline but not serotonin level in pre-synapse (33). Another study suggested that lower dose of VLX (15 mg/kg) was not able to elicit 5-HT1A receptors-stimulated G protein activation (34). Levels of noradrenaline are obviously lowered in several areas in older mice brains, including the hypothalamus (35). In human, reduction in noradrenergic-dependent functions in locus coeruleus is associated with impaired cognitive and behavioral function (36). Based on the above previous reports and our findings here, we assumed that the beneficial effects of VLX in this mouse model might not be derived from the direct effects on neurotransmitters. Inflammatory responses and immune reaction play role in cognitive decline after surgery (37). Therefore, we wondered whether VLX regulate the inflammatory response in this mouse model. Our assumption was further encouraged by the recent publication that indicated VLX could alleviate the neuroinflammation and depressive-like behaviors in a mouse model of demyelination (31).

It has been well-established that tissue trauma released damage-associated cytokines, including TNF-α, could break the blood–brain barrier (BBB) and result in neuroinflammation and concomitant cognitive decline (38). Moreover, recent studies have demonstrated that anesthetic alone could cause damages to the hippocampus, which led to memory and cognitive dysfunction (3, 39). Therefore, we investigated the pathological hippocampal tissue and the hippocampus related behavioral performance in this mouse model. Current studies of neuropsychiatric diseases, including POCD, were primarily on the disturbance of neurotransmitters, but recent studies emphasized the importance of neuroinflammation (8).

The inhibitory neurotransmitter, GABA, is a prominent player in regulating learning and memory (40). Particularly, the GABA_A_ receptor α5 subunit plays a key role in POCD (3, 7). A previous study also suggested that α5 could actively regulate the memory deficits induced by inflammation (41). Therefore, we postulated that preventing the ISO-induced activation of α5 could inhibit the inflammation as well. STAT3 is a key player in regulating inflammatory gene expression and glial reactivity in CNS to various insults and stimulations (42). Specifically, JAK2/STAT3 signaling pathway was involved in POCD in rat model by increasing their phosphorylation, and inhibiting the levels of p-JAK2 and p-STAT3 could attenuate the symptoms of POCD in rats (43). A study has shown that VLX possesses anti-inflammation property (44). Consistent with the previous findings, we demonstrated that VLX could inhibit the expression of p-STAT3 and reduce the expression of some cytokines (Figure 4). It has been well-established that VLX could protect neurons against insults in CNS. Combined with anti-inflammation effect, VLX might exert therapeutic effects by targeting multiple ways in the CNS to prevent the memory and cognitive loss in POCD. Impaired spatial learning memory caused by ISO has been reported (45). Neuroinflammation could be attributed to the development of the deficits (45). Studies have indicated that anesthetics alone could cause POCD in several models (3, 46). A study also suggested that ISO caused POCD via triggering neuroinflammation (46). Our study further confirmed this property of ISO in a mouse model. More importantly, here, we demonstrated an effective method to rescue these mice from the neuronal damage on the behavioral level. The most important finding in our study was the molecular mechanism by which VLX prevented the surface expression of one of GABA_A_ receptors, α5. The persistent increased surface expression of this receptor has been shown to be closely associated with the increase in tonic current, which might eventually cause the memory and cognitive impairment of these mice (3). Here, we found that ISO could induce the surface expression of α5 in mice, and the increased expression could be modulated by the treatment of VLX. These findings were consistent with previous reports. More importantly, our results indicated that VLX, an available compound, could prevent the increased surface expression of α5 that was validated to be associated with the POCD (3). Accordingly, we found the VLX treatment could effectively ameliorate the impairment of working memory induced by ISO in a Y maze test. Since there is no report of the direct effect of VLX on the GABA_A_ receptors, the indirect regulation of VLX on α5 subunit is quite possible. As a typical serotonin–norepinephrine reuptake inhibitor (SNRI) antidepressant, VLX has potent effect on serotonin receptor and increase the content of serotonin. Serotonin has been found to be a modulator of GABA neurotransmission (47), and therefore, serotonin receptor may mediate the effect of VLX to GABA_A_ receptors α5 subunit.

VLX possesses obvious therapeutic effect on mood disorders, and meanwhile, it exerts anti-inflammatory effect in CNS (48). A study has indicated that VLX was able to exert obvious neuroprotective effects in brain injuries (17). In addition, STAT3 activation is essential for neuronal regeneration (49). By working on both neurotransmitters and inflammation in CNS, VLX may open a new road to explore the treatment options. Although multiple factors may predispose POCD, only aging is identified as an important one so far (50). Therefore, we admitted the limitations of the present study such as the young age of mice, which was not the best scenario for mimicking the patients clinically since POCD occurs more often in the elderly patients. However, young mice are more suitable for further cellular and molecular mechanism study (3, 50, 51). Old mice were usually used to compare the age effects on the mice response (39). Future studies involving the relative old mice are warranted for this purpose. In addition, memory and cognitive dysfunctions induced by anesthetics could be affected by several factors, including treatment protocol (anesthetic alone or combining with surgical procedure) and the age of mouse used in the studies. For example, a previous study found that ISO exposure could affect acquisition in a water maze test but influence the retention in young not older rats (52). In the present study, we attempted to explore the effects of ISO on the reference memory of mice by carrying out a water maze test. We found under our specific treatment protocol that there was no significant difference in either acquisition or retention trials in water maze test among the control and ISO treatment group (Supplementary Figure 1). To further confirm the protective effects of VLX in the mouse model, efforts on other behavioral assays of the memory and cognitive function should be put on future studies, such as puzzle box test, which was recently claimed by a research group with using the etomidate in a mouse model of POCD (7).

## Data Availability Statement

The original contributions presented in the study are included in the article/Supplementary Material, further inquiries can be directed to the corresponding author/s.

## Ethics Statement

The animal study was reviewed and approved by The Experimental Animal Ethics Committee of China-Japan Union Hospital of Jilin University, Jilin Province, China.

## Author Contributions

LL designed the study, wrote the draft, and finalized the manuscript. CZ performed the assays and edited the manuscript. LL was a visiting scholar in CZ's team (Department of Thyroid Surgery, China–Japan Union Hospital of Jilin University, Changchun, Jilin Province, China). Both authors contributed to the article and approved the submitted version.

## Conflict of Interest

The authors declare that the research was conducted in the absence of any commercial or financial relationships that could be construed as a potential conflict of interest.
