# Supplementary material for: Venlafaxine Attenuated the Cognitive and Memory Deficit in Mice Exposed to Isoflurane Alone
Source: Front Neurol. 2021 Feb 23;12:591223. doi: 10.3389/fneur.2021.591223 (PMC7940694; doi:10.3389/fneur.2021.591223)
Supplement: Supplementary file 1 [file Image_1.pdf]

Supplementary Figure 1

Water maze assay (*Glia. 2016, 64(2): 240-54*) was performed 3 days after ISO treatment as we did with Y maze. There was no obvious difference between control and ISO treatment groups on the escape latency for locating the platform and the probe test in the last day to locate the quadrant where the platform was before. As shown in the figures, there were no obvious changes of memory on training stages (**A**, 4 days) and the probe stage (**B**, 5th day of the test).

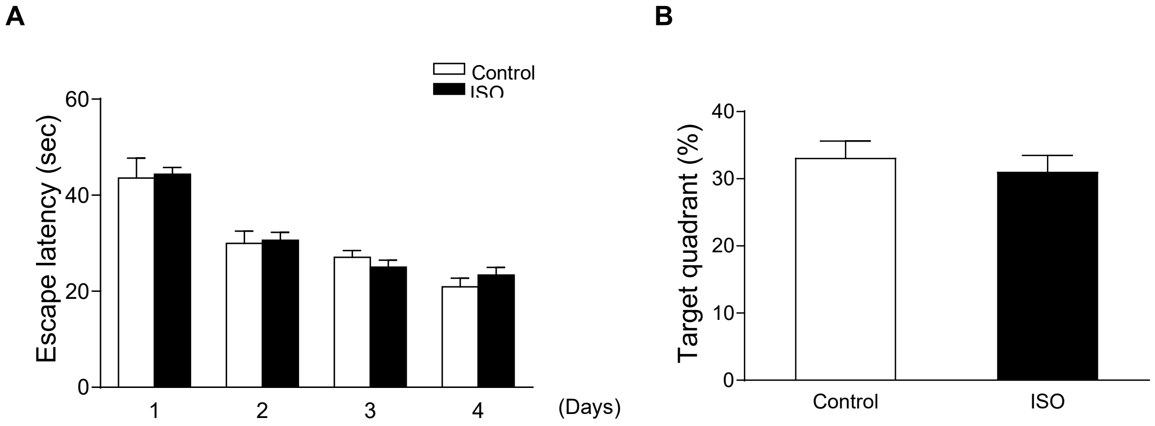

(**A**) Escape latency of mice in the hidden-platform test. (**B**) Percentage of time spent searching for the target quadrant in the probe test. All data are expressed as means ± SEM. n=10.
